# A new ferroptosis-related signature model including messenger RNAs and long non-coding RNAs predicts the prognosis of gastric cancer patients

**DOI:** 10.2478/jtim-2023-0089

**Published:** 2023-07-05

**Authors:** Yang Liu, Yanqing Liu, Shujun Ye, Huijin Feng, Lianjun Ma

**Affiliations:** Endoscopy Center, China-Japan Union Hospital of Jilin University, Changchun 130033, Jilin Province, China; Herbert Irving Comprehensive Cancer Center, Columbia University, New York 10032, NY, USA

**Keywords:** gastric cancer, ferroptosis, long non-coding RNA, messenger RNA, tumor biomarkers, prognostic model

## Abstract

**Background and Objectives:**

Gastric cancer (GC) is among the most malignant tumor types, which causes heavy healthy and economic burden to the people and societies all around the world. Establishment of an effective set of prognostic marker will benefit a lot to the treatment of GC patients clinically. Ferroptosis is a newly identified regulated cell death modality, with tight relevance with GC development. However, its application in the prognosis of GC has not been studied in detail. Deregulated messenger RNA (mRNA) and long non-coding RNA (lncRNA) expression profile in tumor can serve as novel prognostic marker for predicting the survival and cancer relapse in patients.

**Methods:**

We downloaded ferroptosis-related gene expression microarray data, clinicopathologic information and a list of 259 ferroptosis-related genes from The Cancer Genome Atlas (TCGA), Gene Expression Omnibus (GEO), and Ferroptosis database, respectively. Then, correlation analysis, univariate and multivariate Cox regression analysis were used to construct a novel prognostic model for GC. Then, we validated the model in the GEO datasets. Finally, we evaluated the differences in immune microenvironment between high- and low-risk groups.

**Results:**

We utilized the ferroptosis-related mRNA and lncRNA profile to successfully construct a prognostic model (incorporating 2 mRNAs and 15 lncRNAs) in GC. Our model, integrating diverse clinical traits and critical factors of GC, showed desirable efficacy in the prognosis of GC patients. This model also manifested effectively in validation by using external patients’ data.

**Conclusions:**

Our study developed a novel ferroptosis-related signature to predict the prognosis of gastric cancer patients. The ferroptosis-related signature had a favorable predictive ability. This model may greatly boost the treatment of GC patients in clinical practice.

## Introduction

Gastric cancer (GC) is a common malignant tumor of the digestive tract, with a high incidence rate, high mortality, and poor prognosis. Globally, GC ranked fourth in mortality and fifth in incidence among all cancer types in 2020.^[1]^ GC is a heterogeneous disease related to different causes and mechanisms of carcinogenesis, resulting in the diversity of GC indicators including race, gender, and geographic location.^[2]^ Despite the tremendous progress of treatment in the past decades, the 5-year survival rate of GC is still not ideal.^[3]^ One reason for this is lack of sensitive and effective markers for evaluating the efficacy of therapeutics and predicting the recovery or recurrence of GC patients. Identifying effective prognostic biomarkers is of vital significance for the treatment of GC patients.

Ferroptosis is an iron-dependent regulatory cell death process that is caused by the accumulation of intracellular iron and peroxidation of membrane lipid.^[4,5]^ Although the exact mechanism and physiological function of ferroptosis have not been totally elucidated, more and more evidence shows that it is closely related to various pathological conditions, particularly cancers.^[4,6]^ For GC, ferroptosis has been demonstrated to be an effective way to eradicate the tumor cells.^[7]^ The induction and modulation of ferroptosis involve several key tumor suppressors and oncogenes in cancer metabolism.^[8,9]^ These ferroptosis-related genes may serve as prognostic markers in GC.

Long noncoding RNA (lncRNA) is a kind of special noncoding RNA with a length of more than 200 nucleotides.^[10]^ It is reported that lncRNAs are involved in a wide range of biological processes (BPs), including cell migration, invasion, proliferation, and apoptosis.^[11]^ Dysregulated lncRNA expression profiles are found in various cancer types, indicating that abnormal lncRNA expression may be an important factor in tumorigenesis.^[12,13]^ The lncRNA is also tightly related to the initiation and development of GC.^[14]^ In addition, according to many recent studies, lncRNAs also function as epigenetic regulators in ferroptosis.^[15]^ Attributing to the progress of RNA sequencing technology, lncRNA expression profile has been applied effectively in the prognosis of distinct cancers, including GC.^[16]^ Whether ferroptosis-related lncRNAs can serve as prognostic biomarkers in GC needs to be investigated in depth.

In this study, based on the differentially expressed genes (DEGs) associated with ferroptosis, we developed a prognostic model for GC, which was composed of two messenger RNAs (mRNAs) and 15 lncRNAs. We comprehensively evaluated our model in different clinical settings and validated it in an external dataset. Our model exhibits good performance for the prognosis of GC patients.

## Methods

### Dataset

The fragments per kilobase million reads (FPKM) data and clinicopathologic information of GC were downloaded from The Cancer Genome Atlas (TCGA; http://cancergenome.nih.gov) database, and this data contains 375 tumor samples and 32 normal samples. Gene expression microarray data and clinicopathologic information of GSE84437 were downloaded from Gene Expression Omnibus (GEO; https://www.ncbi.nlm.nih.gov/geo/). A list of 259 ferroptosis-related genes was downloaded from the ferroptosis database (FerrDb; http://www.zhounan.org/ferrdb/operations/download.html).

### Identification of ferroptosis-related DEGs

The ferroptosis-related lncRNAs were derived through correlation analysis between the expression levels of ferroptosis-related genes and lncRNAs (|spearman correlation (cor)| > 0.60, *P* < 0.001). Using *P* < 0.05 and |log^2^(fold change)| (logFC) > 1 as the screening criteria, we obtained ferroptosis-related DEGs including mRNAs and lncRNAs.

### Functional enrichment analysis of DEGs

To understand the BPs of DEGs, we performed Gene Ontology (GO) enrichment analysis and Kyoto Encyclopedia of Genes and Genomes (KEGG) pathway analysis for these DEGs. The criterion for significance was set to *P* < 0.05.

### Construction of a novel prognostic model correlated to GC

To identify survival-related genes, univariate Cox regression analysis was performed first with *P* > 0.05. Then, multivariate Cox regression analysis was used to determine the optimal genes for the construction of a prognostic model. The risk score formula is as follows: risk score = (exprgene1 × Coefgene1) + (exprgene2 × Coefgene2) + 1/4 + (exprgenen × Coefgenen).

The formula was used to calculate the risk score of each GC patient.

### Development of a nomogram

We constructed a model based on the results of the multivariate Cox model. To establish the nomogram model, the R package “rms” was used (R Core Team, Auckland, New Zealand).

### Validation of ferroptosis-related risk model in the GEO dataset

(1) Spearman correlation analysis was used to analyze the correlation between 17 signature genes and mRNAs. Signature genes-related mRNA was defined as 17 signature genes significantly related to at least one signature gene (|cor| > 0.40, *P* < 0.001).

(2) Differential expression analysis was performed between low- and high-risk groups to classify mRNAs into gene clusters A/ B.

(3) Gene set variation analysis (GSVA) was performed for each sample to calculate the enrichment score of the gene clusters A/ B. Next, the score defined as Substitute Score (SS score; a substitute for risk score) is equivalent to the enrichment score of gene cluster A minus the enrichment score of gene cluster B.

(4) By calculating the SS score in each GEO sample, the Kaplan-Meier (KM) analysis method was used to compare the overall survival (OS) between the low SS score group and the high SS score group.

**Figure 1:**
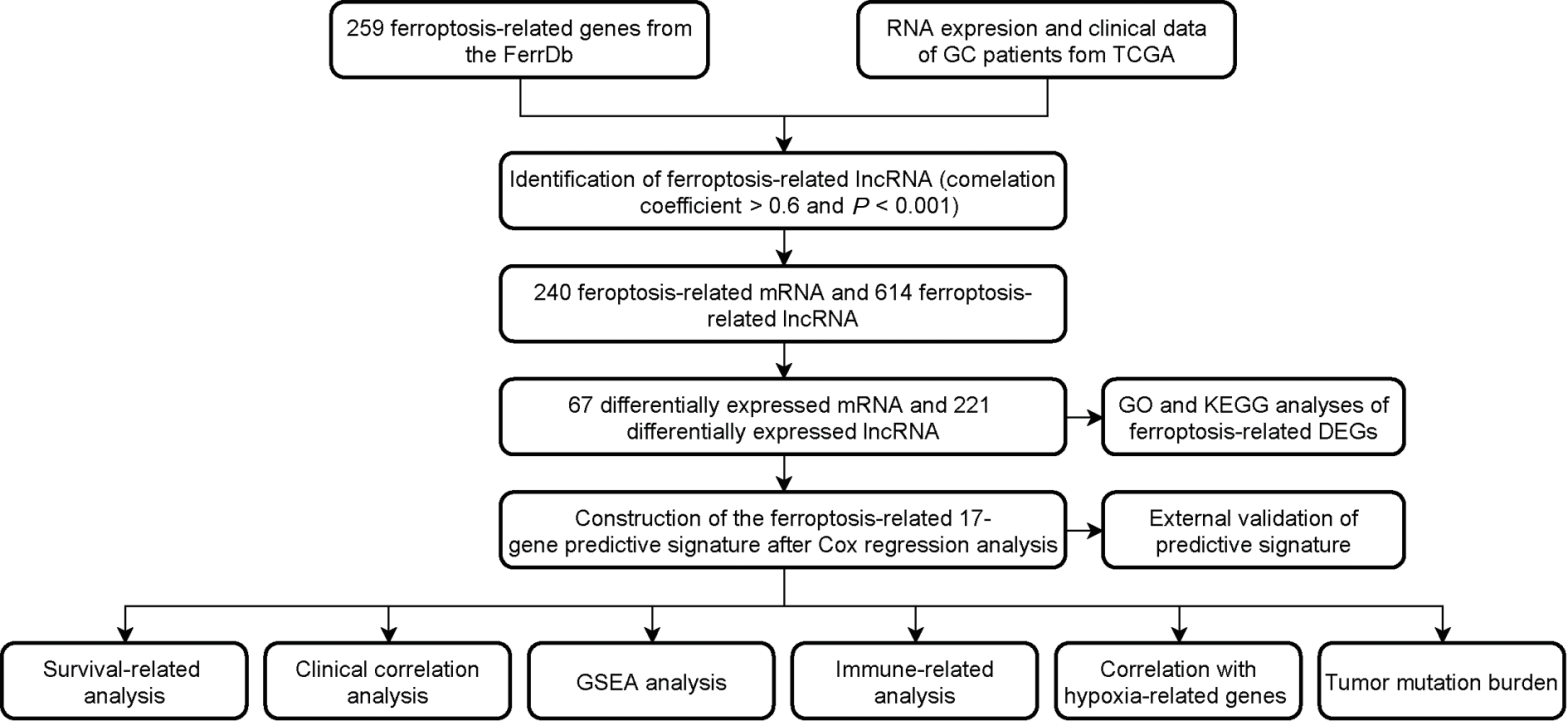
Workflow of the study design. FerrDb: ferroptosis database; GC: gastric cancer; TCGA: The Cancer Genome Atlas database; lncRNA: long non-coding RNA; mRNA: messenger RNA; GO: Gene Ontology; KEGG: Kyoto Encyclopedia of Genes and Genomes; GSEA: gene set enrichment analysis; DEGs: differentially expressed genes.

### Co-expression network analysis of lncRNAs

To explore the relationship between these ferroptosis-related lncRNAs, the co-expression network was constructed based on the results of the previous Pearson correlation analysis.

### Functional analysis

To investigate the potential altered pathways associated with the risk score, the Java software gene set enrichment analysis (GSEA; http://software.broadinstitute.org/gsea/index.jsp) was used for the high- and low-risk groups. The significant pathways are defined with normalized enrichment score (NES) > 1 & NES < −1 and *P* < 0.05.

### Correlation analysis between high- and low-risk groups and clinical traits

In order to further explore the relationship between high-and low-risk groups and clinical traits in GC patients, the differences in tumor nodes metastases (TNM) stage, gender, age, and grade were compared between the high-and low-risk groups.

### A comprehensive evaluation of the immune microenvironment

First, we used different algorithms to calculate immune cell abundance in high- and low-risk groups. In addition, the differences in immune function and immune checkpoints between the two groups were also studied.

## Results

### Identification of ferroptosis-related DEGs

**Figure 2:**
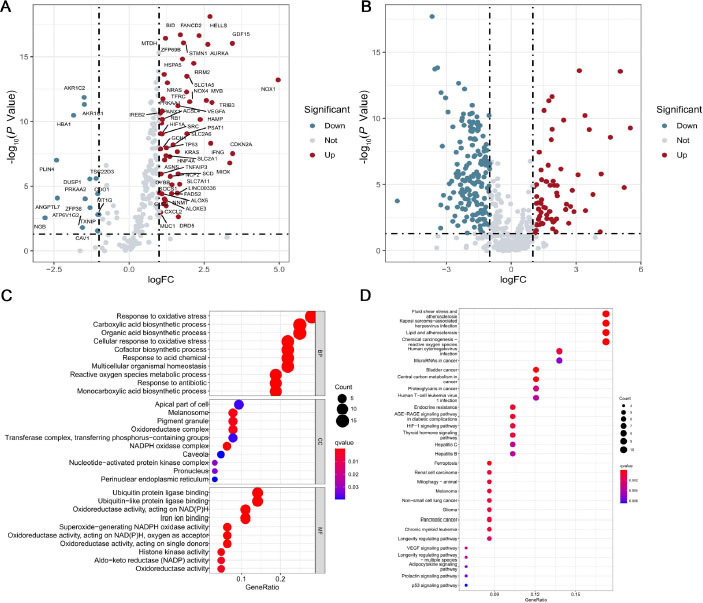
Screening of ferroptosis-related differentially expressed genes. (A) Volcano map of differentially expressed mRNAs. (B) Volcano map of differentially expressed lncRNAs. (C) Results of the GO analysis including cellular component, molecular function, and biological process. (D) Results of the KEGG analysis. GO: Gene Ontology; KEGG: Kyoto Encyclopedia of Genes and Genomes; lncRNAs: long noncoding RNAs; mRNAs: messenger RNAs.

The flowchart of the research is shown in Figure 1. First, the TCGA Stomach Adenocarcinoma (TCGA-STAD) dataset was obtained from the TCGA database via cBioPortal, and the list of 259 ferroptosis-related genes was downloaded from FerrDb. Two hundred and forty ferroptosis-related mRNAs were extracted from the list of ferroptosis-related genes. Next, 614 ferroptosis-related lncRNAs were identified through Pearson correlation analysis of the lncRNAs from the TCGA-STAD dataset and the 259 ferroptosis-related genes (|cor| > 0.60, *P* < 0.001). In the final step, the differential expression analysis of 375 tumors and 32 normal TCGA-STAD samples yielded a total of 288 ferroptosis-related DEGs including 67 mRNAs and 221 lncRNAs (Figure 2A and 2B).

### Functional enrichment analysis of DEGs

In order to further analyze the DEGs, we performed GO analysis and KEGG pathway analysis. GO enrichment analysis includes a cellular component (CC), BP, and molecular function (MF) (Figure 2C). In the BP category, the DEGs were involved in response to oxidative stress, carboxylic acid biosynthetic process, organic acid biosynthetic process, and cellular response to oxidative stress, consistent with the fact that oxidative stress and ferroptosis are closely related. In the CC category, apical part of the cell, melanosome, pigment granule, oxidoreductase complex, and so on were significantly enriched. In the MF category, the DEGs were enriched in ubiquitin proteinligase binding, ubiquitin-like protein ligase binding, and oxidoreductase activity, acting on reduced nicotinamide adenine dinucleotide phosphate (NADPH) and iron ion binding, and so on. KEGG pathway analysis showed that the ferroptosis-related DEGs were mainly enriched in fluid shear stress and atherosclerosis, lipid and atherosclerosis, advanced glycation end products (AGE)-receptor for advanced glycation end products (RAGE) signaling pathway in diabetic complications, Kaposi sarcoma-associated herpesvirus infection, hypoxia inducible factor-1 (HIF-1) signaling pathway, and so on (Figure 2D). Furthermore, the lipid and atherosclerosis and the HIF-1 signaling pathways are worth noting. HIF-1 is a heterodimeric protein which is composed of two proteins HIF-1α and HIF-1β.^[17]^ Previous studies have shown that hypoxia and hypoxia-related signaling pathways play an important role in the occurrence and development of GC.^[18, 19, 20]^

### Construction of a novel prognostic-predicting model correlated to GC

**Figure 3:**
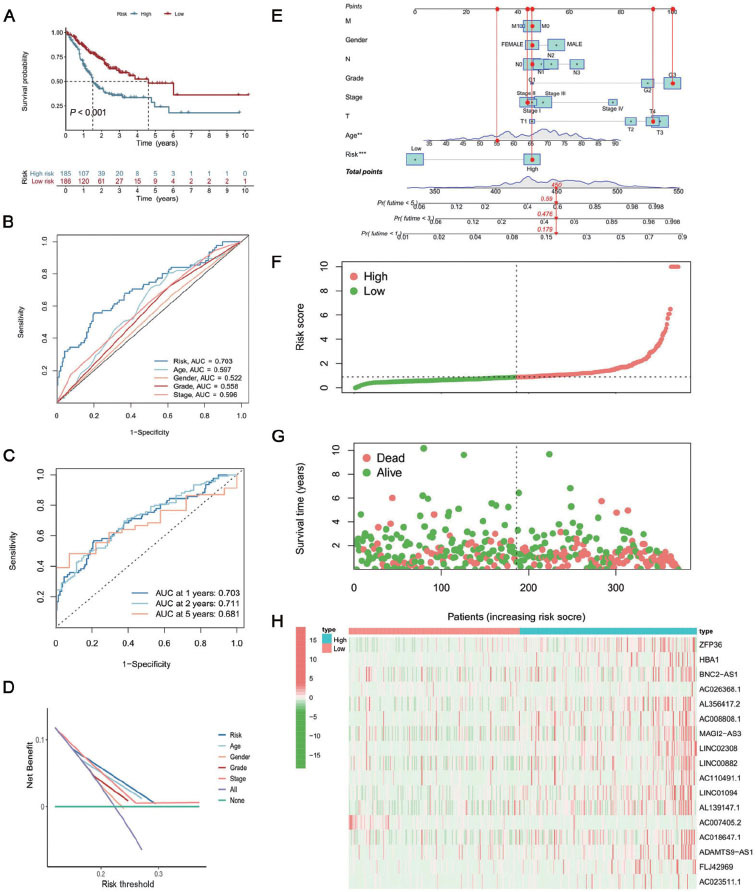
Establishment and evaluation of the prognostic model. (A) Results of Kaplan-Meier analysis and the log-rank test in the different risk groups. (B) Time-dependent ROC curve of the risk score at 1, 2, and 5 years. (C) ROC curves of a risk score for clinical features in GC patients. (D) DCA of the risk factors and clinical traits. (E) The nomogram combining clinical traits and risk score predicts 1-, 3-, and 5-year OS of GC patients. (F) Risk score distribution of patients with GC. (G) Survival status distribution of patients with GC. (H) The expression heatmap of these mRNAs and lncRNAs. DCA: decision curve analysis; GC: gastric cancer; lncRNAs: long noncoding RNAs; mRNAs: messenger RNAs; OS: overall survival; ROC: receiver operating characteristic; AUC: area under the receiver operating characteristic curve. **P < 0.01; ***P < 0.001

Next, we studied the relationship between ferroptosis-related DEGs and GC patient survival. In the univariate Cox regression analysis, 52 DEGs were significantly associated with OS. Then, the multivariate regression model was established by using the multivariate Cox stepwise regression method. Multivariate Cox regression analysis showed that 17 ferroptosis-related DEGs including two mRNAs and 15 lncRNAs were identified to construct a prognostic-predicting model. The correlation between the levels of these 15 lncRNAs and ferroptosis genes is shown in Figure 3. The formula used to calculate the risk score of the 17 signature genes was as follows: risk score = (ZFP36 expression) × (0.000586561) + (HBA1 expression) × (0.009743716) + (BNC2-AS1 expression) × (–0.002581118) + (AC026368.1 expression) × (0.000249402) + (AL356417.2 expression) × (0.003059539) + (AC008808.1 expression) × (0.008982071) + (MAGI2-AS3 expression) × (–0.003468585) + (LINC02308 expression) × (0.017145294) + (LINC00882 expression) × (–0.014345102) + (AC110491.1 expression) × (0.010071511) + (LINC01094 expression) × (0.001808894) + (AL139147.1 expression) × (0.007782626) + (AC007405.2 expression) × (–0.004812248) + (AC018647.1 expression) × (0.016655829) + (ADAMTS9-AS1 expression) × (0.007527902) + (FLJ42969 expression) × (0.004832929) + (AC023511.1 expression) × (0.001121488). KM analysis revealed that the OS in the low-risk group was significantly higher than that in the high-risk group (Figure 3A). Model evaluation predictive accuracy was assessed by the area under the receiver operating characteristic curve (AUC), and the AUCs at 1, 2, and 5 years were 0.703, 0.711, and 0.681, respectively (Figure 3B). To further evaluate the susceptibility and uniqueness of the risk score, we calculated the risk score and other clinical factors (such as age, gender, and grade) (Figure 3C). The AUC for the risk score was significantly greater than the AUC for other clinical factors, proving that the risk level had a better predictive value. Decision curve analysis (DCA) further showed that the risk score was a more accurate indicator than other clinical traits (Figure 3D). According to the distribution plot of the risk score and survival status, the higher the risk score, the higher the number of deaths of GC patients. Consecutively, we constructed a nomogram with clinical traits and the risk score to predict the 1-, 3-, and 5-year prognosis of GC patients (Figure 3E). The distribution of the patient’s risk score, survival status, and survival time in the TCGA-STAD is presented in Figure 3F–3H.

### Validation of ferroptosis-related risk model in GEO dataset

**Figure 4:**
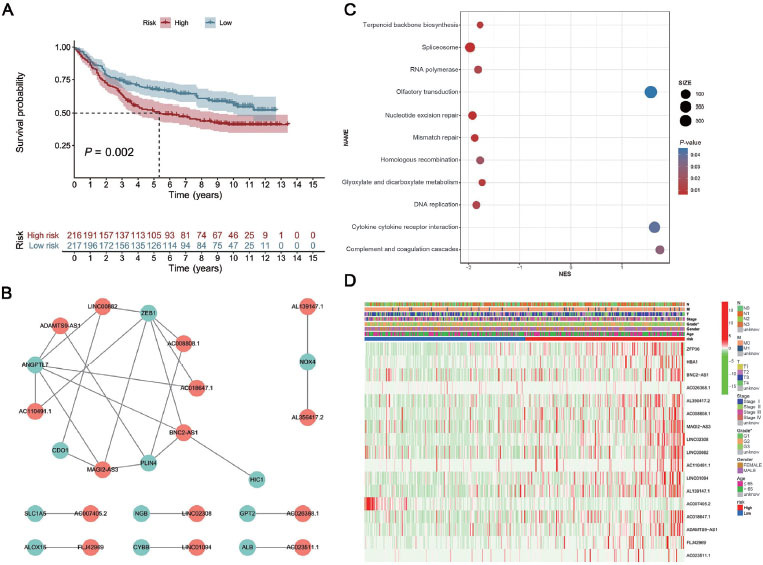
Biological characteristics of the different risk groups in GC patients. (A) Results of Kaplan-Meier analysis and the log-rank test for the different SS score groups in the GEO dataset. (B) The co-expression network of lncRNA–mRNA. (C) Results of the GSEA. (D) Heatmap of the ferroptosis-related genes’ prognostic signature and clinical traits in GC patients. GC: gastric cancer; GEO: Gene Expression Omnibus; GSEA: Gene Set Enrichment Analysis; lncRNA: long noncoding RNA; mRNA: messenger RNA; SS score: Substitute Score.

It is difficult to validate the performance of the risk model since no corresponding lncRNAs exist in other datasets. Therefore, we studied the prognostic value of the SS score in TCGA-STAD and GSE84437. There was a significant correlation between the risk score and the SS score in TCGA-STAD (*P* = 2.2 × 10^–16^). The above analysis shows that the risk score is a reliable indicator. KM analysis revealed that the risk mode was related to the survival status of patients (*P* < 0.005) (Figure 4A).

### Co-expression network analysis of ferroptosis-related lncRNAs

In order to deepen the understanding of the potential interactions of the 17 signature genes, we derived 12 related genes by gene co-expression analysis. Cytoscape software was used to construct the lncRNA–mRNA co-expression network diagram (Figure 4B). Seven lncRNAs (LINC008, AC008808.1, *etc*.) and five genes (zinc finger E-box binding homeobox 1 [*ZEB1*], angiopoietin like 7 [*ANGPTL7*], *etc*.) were assigned to the same module.

### Functional analysis

We used GSEA to study the potential biological functions of the 17 signature genes (Figure 4C). The results of GSEA showed that there are many tumor-related pathways in high-risk groups, including olfactory transduction, cytokine–receptor interaction, and complement and coagulation cascade. Meanwhile, the low-risk group was enriched in cell cycle-related pathways such as mismatch repair, DNA replication, and homologous recombination.

### Correlation analysis between high- and low-risk groups and clinical traits

Cluster analysis of clinical traits (such as grade, TNM stage, age, and gender) and target genes showed that the clinical grade of patients in the high-risk group was later than that in the low-risk group (Figure 4D).

### A comprehensive evaluation of the immune microenvironment

**Figure 5:**
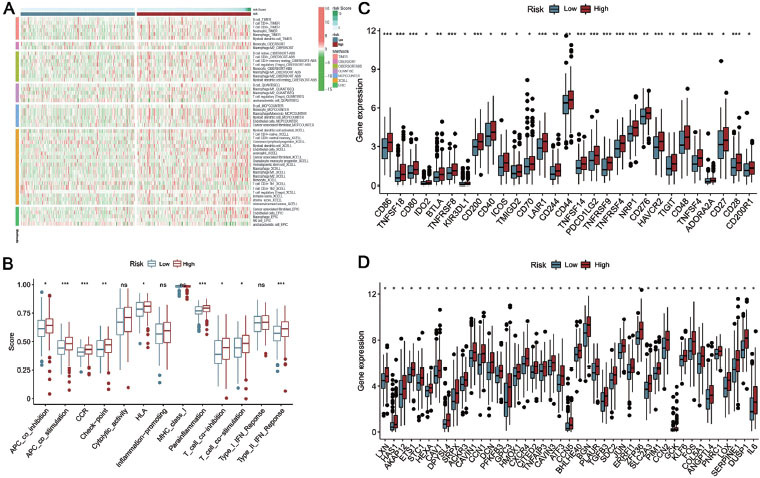
Differences in immune status between high- and low-risk GC patients. (A) The landscape of immune infiltration in high- and low-risk GC patients. (B) Comparison of immune-related functions between the high- and low-risk groups in GC patients. (C) Comparison of immune checkpoints between the high- and low-risk groups in GC patients. (D) Comparison of the expression of hypoxia-associated genes between the high- and low-risk groups. *P < 0.05; **P < 0.01; ***P < 0.001. GC: gastric cancer.

The microenvironment has a significant impact on tumor development. To explore the relationship between the risk score and immune cell infiltration, we conducted correlation analyses using multiple algorithms (Figure 5A). The results showed that there were significant differences in immune response between the high- and low-risk groups. In addition, in terms of immune function, the high-risk group was significantly stronger than the low-risk group (Figure 5B). Subsequently, we compared the immune checkpoint molecules between the two groups. The results showed that all the checkpoint molecules had higher expression in the high-risk group (Figure 5C). The above analysis shows that the high-risk group has a higher immune activity as a whole. Therefore, the immunotherapy response rate of the high-risk group classified by our risk signature may have a potentially higher response rate than the low-risk group.

### Relationship between hypoxia-related gene and risk score

Recent research suggested a close relationship between hypoxia and ferroptosis.^[21]^ Therefore, we investigated the expression of hypoxia-related genes between the low- and high-risk groups (Figure 5D), and the results showed that the expression of many hypoxia-related genes (such as latexin, hyaluronan synthase 1 [*HAS1*], A-kinase anchor protein 12 [*AKAP12*], *etc*.) in the high-risk group was obviously higher (*P* < 0.001) than that in the low-risk group. In combination with the above results, such as enrichment of HIF-1 signaling pathway in enrichment analysis results and susceptibility of the high-risk group to the c-Jun N-terminal kinase (JNK) inhibitor, we think the relationship between ferroptosis and hypoxia in GC should be paid more attention in further studies.

### Relationship between tumor mutation burden and the risk score

**Figure 6:**
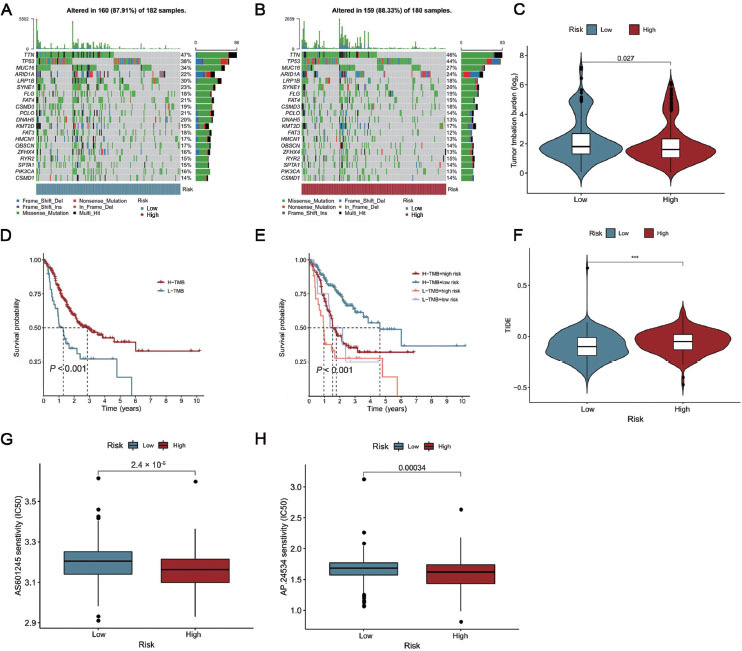
Mutation landscape of the different risk groups in GC patients. (A, B) The oncoplots of the somatic mutation in the high- and low-risk groups in GC patients. (C) Violin plot for the TMB scores between the high- and low-risk groups in GC patients. (D) The Kaplan-Meier curve was used to analyze the survival of patients with low TMB and high TMB. (E) The Kaplan-Meier curve was used to analyze survival of the subgroup of patients stratified by both risk score and TMB. (F) Violin plot for the TIDE scores between the high- and low-risk groups in GC patients. (G) AS601245 sensitivity analysis between the high- and low-risk groups in GC patients. (H) AP.24534 sensitivity analysis between the high- and low-risk groups in GC patients. ***P < 0.001. GC: gastric cancer; TMB: tumor mutation burden; TIDE: tumor immune dysfunction and exclusion.

Tumor mutation burden (TMB) refers to the total number of nonsynonymous variants or single nucleotide variants in each tumor genome region.^[22]^ To understand the relationship between the risk score and gene mutation, simple nucleotide variation data was downloaded and analyzed from TCGA. Figure 6A and 6B shows a summary of gene mutation information. Tumor protein p53 (*TP53*) (or *p53*) and mucin-16 (*MUC16*) were the most common mutant genes in the two groups. *P53* is also a crucial tumor suppressor and a master regulator of ferroptosis.^[9,23,24]^ The mutation rate of p53 was 6% higher and that of low-density lipoprotein (LDL) receptor-related protein 1B (*LRP1B*) was 12% lower in the high-risk group compared to the low-risk group. As observed in Figure 6C, the high-risk score group showed lower TMB than the low-risk group. GC patients with high levels of TMB had a better prognosis than the patients with low levels of TMB (Figure 6D). In addition, we also combined the risk score and TMB level to predict the prognosis of GC. Interestingly, we found that GC patients with a high TMB level and low risk score had the best prognosis (Figure 6E). As shown in Figure 6F, the low-risk group may be more likely to activate the immune response, and patients in the low-risk group may be more sensitive to immune checkpoint therapy. Subsequently, the sensitivity of high- and low-risk groups to common chemotherapeutic drugs was evaluated by half-maximal inhibitory concentration (IC50) value. The IC50 value of patients in the high-risk group was significantly lower, and they were more sensitive to AP.24534 (ponatinib) and AS601245 (a JNK inhibitor) (Figure 6G and 6H).

***Comparison with other published gene signatures*** We compared our 17 gene signatures with other published gene signatures (Table S1).^[25, 26, 27, 28, 29]^ First, an important advantage of our model was that our model combined lncRNA and mRNA. Next, when ferroptosis-related lncRNAs were identified based on the Pearson correlation, screening conditions in our model (|cor| > 0.6, *P* < 0.001) were the strictest among these models. Strict screening conditions ensured strong correlations between screened lncRNA and ferroptosis. As shown in Table S1, the 1-year AUC in our 17-gene signature was 0.703 and the 5-year AUC was 0.681, and the results were verified using external validation. The AUCs of three-gene of Zhang, four-gene of Cai, 20-gene of Chen, 17-gene of Pan, and four-gene of Wei were 0.756, 0.638, 0.830, 0.751, and 0.636, respectively, and the majority of other studies only verified the results in internal data sets. Compared to the other five published gene signatures, our model had an excellent prognostic potential.

## Discussion

GC is the most common malignant tumor of the digestive system.^[30]^ Due to the lack of evident symptoms and early screening methods, the diagnosis of GC at an early stage is difficult, resulting in a poor prognosis. Although the therapeutic effects of various treatments of GC have improved in recent years, the rate of morbidity due to GC remains high.^[31]^ Therefore, exploring new and feasible prognostic biomarkers is very important to improve the prognosis of GC patients. In this context, the present study aimed to develop a novel molecular signature based on ferroptosis-related lncRNA and mRNA in GC.

The prognostic model constructed in this study contained 17 genes, including 15 lncRNAs (BNC2-AS1, AC026368.1, AL356417.2, AC008808.1, MAGI2-AS3, LINC02308, LINC00882, AC110491.1, LINC01094, AL139147.1, AC007405.2, AC018647.1, ADAMTS9-AS1, FLJ42969, AC023511.1) and two mRNAs (zinc finger protein 36 homolog [*ZFP36*] and hemoglobin subunit α1 [*HBA1*]). ZFP36 encoded by *ZFP36* gene is also known as tristetraprolin (TTP).^[32]^ ZFP36 acts through binding to the 3'-untranslated region (3'-UTR) and recruitment of deadenylation and degradation factors to decrease the stability of target mRNA, such as tumor necrosis factor (TNF), interleukin-6 (*IL-6*), interleukin-17A (*IL-17A*), and interleukin-33 (*IL-33*).^[32]^ ZFP36 can alter the response of cells to lipid peroxidation, oxidative stress, apoptosis, and immune stimulation through its posttranscriptional effect on the target mRNA.^[33]^ In addition, ZFP36 has been considered to be associated with ferroptosis.^[34]^ There were also studies suggesting that ZFP36 is relevant to several types of tumors including GC.^[35]^ The *HBA1* gene provides instructions for making a protein called α-globin, and α-globin is a component (subunit) of a larger protein called hemoglobin, which is the protein in red blood cells that carries oxygen. Studies have shown that stimulating ferroptosis may trigger the differential expression of *HBA1*, and *HBA1* might affect ferroptosis by influencing phosphoserine aminotransferase 1 (*PSAT1*) activity.^[36]^ Previous studies have also reported *HBA1* is upregulated in GC as an antioxidant-related gene.^[37]^

Among the genes with the correlation coefficient in our risk model, LINC02308 was reported to exert its carcinogenic effect by binding to mir-30e-3p.^[38]^ AC110491.1 was reported to be associated with endometrial carcinoma and bladder cancer.^[39,40]^ ADAMTS9-AS1 was reported to be involved in many tumors, including breast cancer, glioma, lung cancer, liver cancer, and so on.^[41,42]^ AL356417.2 has been recently reported to be associated with predicting survival and immune infiltrating status in breast cancer.^[43]^ Numerous studies have shown that LINC01094 is correlated with the prognosis of many tumors, such as ovarian cancer, renal cell carcinoma, pancreatic cancer, and others.^[44,45]^ It has been suggested that AC026368.1 is involved in the process of autophagy in GC.^[46]^ As the gene with the most negative correlation coefficient in our risk model, LINC00882 is abnormally expressed in several tumors.^[47,48]^ MAGI2-AS3, widely expressed in human cancers, is related to the progression and prognosis of cancer.^[49]^ BNC2-AS1 has been reported to have effects on the proliferation, migration, and invasion of GC cells.^[50]^ However, there are only a few studies on AC018647.1, AC008808.1, AL139147.1, FLJ42969, AC023511.1, and AC007405.2; therefore, further research is needed to figure out their roles in cancers, particularly GC. In summary, in our model, these above-mentioned mRNAs and lncRNAs act together effectively as prognostic markers for GC patients.

There exist several limitations in the research. First, we still need more samples to test the applicability of predictive signatures. Particularly, external data from large-scale multicenter cohorts would help to validate and improve our model. Next, we need to conduct functional experiments in the laboratory to verify the findings and clarify the role of the lncRNAs and mRNAs in GC. In addition, an interesting finding in our study that the ferroptosis and hypoxia are related in GC needs to be investigated further. Combined with the experimental data, our signature can serve better in clinical use.

In conclusion, our study developed a novel ferroptosis-related signature to predict the prognosis of GC patients. The ferroptosis-related signature had a favorable predictive ability. It can be used to calculate the risk score, and it accurately reflects the tumor environment and pharmaceutical landscape; also, it can be used for the prognostic prediction in GC, and thus provides a reference for clinical treatment. Therefore, the signature is expected to become a new biomarker for the diagnosis and treatment of GC patients.

## Supplementary Material

Supplementary Material
