# Supplementary material for: A new ferroptosis-related signature model including messenger RNAs and long non-coding RNAs predicts the prognosis of gastric cancer patients
Source: J Transl Int Med. 2023 Jul 5;11(2):145–55. doi: 10.2478/jtim-2023-0089 (PMC10680379; doi:10.2478/jtim-2023-0089)
Supplement: Supplementary file 1 — Supplementary Material [file jtim-2023-0089_SM.pdf]

Supplementary Material

Supplementary Table 1: Comparison of our risk model and other models

Study	Signature	Screening Criteria	AUC in the training set	Validation
Our study	17-Gene	$\lvert cor \rvert > 0.6$, $P < 0.001$	0.703 (1-year AUC), 0.681 (5-year AUC)	External validation
Zhang *et al*. (2022)[25]	3-Gene	Unknown	0.660 (3-year AUC), 0.756 (5-year AUC)	Internal validation
Cai *et al*. (2022)[26]	4-Gene	$\lvert cor \rvert > 0.3$, $P < 0.001$	0.615 (1-year AUC), 0.638 (5-year AUC)	No Verification
Chen *et al*. (2021)[27]	20-Gene	$\lvert cor \rvert > 0.3$, $P < 0.01$	0.830 (unknown)	Internal validation
Pan *et al*. (2021)[28]	17-Gene	$\lvert cor \rvert > 0.4$, $P < 0.05$	0.751 (unknown)	Internal validation
Wei *et al*. (2021)[29]	4-Gene	$\lvert cor \rvert > 0.4$, $P < 0.001$	0.636 (unknown)	No Verification

AUC: area under the receiver operating characteristic curve.
